# Nicorandil in Patients with Acute Myocardial Infarction Undergoing Primary Percutaneous Coronary Intervention: A Systematic Review and Meta-Analysis

**DOI:** 10.1371/journal.pone.0078231

**Published:** 2013-10-22

**Authors:** Muli Wu, Zheng Huang, Haojun Xie, Zhongjiang Zhou

**Affiliations:** 1 Department of Cardiology, Nanfang Hospital, Southern Medical University, Guangzhou, Guangdong, China; 2 Department of Respiratory and Critical Care Medicine, Nanfang Hospital, Southern Medical University, Guangzhou, Guangdong, China; Scuola Superiore Sant'Anna, Italy

## Abstract

**Background:**

Nicorandil, as an adjunctive therapy with primary percutaneous coronary intervention (PCI), had controversial benefits in cardioprotection in patients with acute myocardial infarction (AMI).

**Methods and Results:**

We performed a systematic review of randomized controlled trials (RCTs) comparing treatment with nicorandil prior to reperfusion therapy with control (placebo or no nicorandil) in patients who suffered from AMI and performed primary PCI. PubMed, EMBASE and CENTRAL databases and other sources were searched without language and publication restriction. 14 trials involving 1680 patients were included into this meta-analysis. Nicorandil significantly reduced the incidence of thrombolysis in myocardial infarction (TIMI) flow grade ≤2 (risk ratio [RR], 0.57; 95% confidence interval [CI]: 0.42 to 0.79), the Timi frame count (TFC) (mean difference [MD], -5.19; 95% CI: -7.13 to -3.26), increased left ventricular ejection fraction (LVEF) (%) (MD, 3.08; 95% CI: 0.79 to 5.36), and reduced the incidence of ventricular arrhythmia (RR, 0.53; 95% CI: 0.37 to 0.76) and congestive heart failure (CHF) (RR, 0.41; 95% CI: 0.22 to 0.75). No difference in the pear creatine kinase (CK) value (MD, -290.19; 95% CI: -793.75 to 213.36) or cardiac death (RR, 0.39; 95% CI: 0.09 to 1.67) was observed.

**Conclusions:**

Nicorandil prior to reperfusion is associated with improvement of coronary reflow as well as suppression of ventricular arrhythmia, and further improves left ventricular function in patients who suffered from AMI and underwent primary PCI. But the definite clinical benefits of nicorandil were not found, which may be due to the small sample size of the selected studies.

## Introduction

Early reperfusion of totally occluded coronary arteries reduces infarct size, cardiac mortality rates, and in-hospital events [1,2]. However, some patients continue to have deteriorating cardiac function and bad prognoses, thought to be due to reperfusion injury. Reperfusion injury probably represents myocyte cell death due to reperfusion, no reflow, myocardial stunning and reperfusion arrhythmias [3]. Nicorandil, a hybrid of an adenosine triphosphate (ATP)-sensitive potassium channel opener and nitrates, was used as an adjunctive therapy with primary percutaneous coronary intervention (PCI) for acute myocardial infarction (AMI). The mechanisms for the salutary actions of nicorandil have been postulated, including anti-free radical and neutrophil modulating properties [4,5], vasodilatation of small coronary arteries [6], and mimicking of ischaemic preconditioning [7]. But relevant clinical trials showed controversial results on whether nicorandil had potential to improve coronary artery reflow and ventricular function [8-10]. In addition, rare events of clinical outcomes were reported because of many trials with limited sample size. Thus we performed a systematic review of randomized controlled trials (RCTs) to investigate the effect of nicorandil prior to reperfusion therapy on cardioprotection and clinical outcomes in patients with AMI.

## Methods

### Data sources and searches

We identified all published studies, including full-text and abstract, which compared the effect of nicorandil by intravenous and/or intracoronary administration with control (placebo or no nicorandil treatment) prior to reperfusion by primary PCI in AMI patients. Searches were performed using Cochrane Central Register of Controlled Trials (CENTRAL) (The Cochrane Library Issue 12, 2012), Pubmed (1966 to December 2012), Embase.com (1974 to November 2012) and ISI meeting and proceedings (previous to November 2012) electronic databases. The search strategy was developed without language and publication restriction and used the medical subject headings and text words, such as “nicorandil”, “myocardial infarction”, and “randomized controlled trials”. We handsearched the supplements of seven international core journals on the cardiovascular field, and manually scanned the reference lists of all eligible articles and relevant meta-analyses. We also searched Google Scholar, TCTMD and www.clinicaltrial.gov websites for unpublished trials (see Table **S1**).

### Studies selection

Two reviewers (M. W. and H. X.) performed study selection independently, with disagreements solved through discussion and by the opinion of a third reviewer (Z. H.) if necessary. Studies were considered potentially eligible for this systematic review if they met the following criteria: (1) RCTs about patients who suffered from AMI and performed primary PCI, (2) nicorandil was administered prior to reperfusion by intravenous and/or intracoronary and compared with control (placebo or no nicorandil treatment), and (3) the studies included at least one of the following interesting outcomes: thrombolysis in myocardial infarction (TIMI) flow grade after PCI, TIMI frame count after PCI, left ventricular ejection fraction (LVEF), peak creatine kinase (CK) value and clinical outcomes including cardiac death, ventricular tachycardia (VT) or fibrillation (VF), or congestive heart failure (CHF)

### Data extraction and quality assessment

Two reviewers (M. W. and H. X.) independently undertook the data extraction and the quality assessment. The disagreements would be solved through discussion and by the opinion of a third reviewer (Z. H.) if necessary. The risk of bias was assessed according to the guidance in the Cochrane Handbook version 5.1.0 [11]. We rated the risk of selection bias by assessing randomization and allocation concealment, of performance bias by assessing blinding of participants and personnel. The risk of detection bias and attrition bias were rated by assessing blinding of outcome assessment for the outcome TIMI flow grade and LVEF, respectively and selection bias for the outcome TIMI flow grade only. We tried to contact the authors by email for obtaining information, if their articles did not report the information in detail. For studies that were reported in > 1 publications, we extracted data from the most complete publication and used other publications as supplements.

### Statistical analysis

The verified data were analyzed using Review-Manager software (RevMan, version 5.1.6 for Windows). We determined pooled mean difference (MD) and corresponding 95% confidence intervals (CI) for continuous data, and pooled risk ratios (RRs) for dichotomous data. If statistically significant difference existed in the endpoints with dichotomous data, we would calculate the number needed to treat (NNT) from the pooled risk difference for each endpoint. The appropriateness of pooling data across studies was assessed with the use of the Cochran Q and the I^2^ test for heterogeneity. Data were pooled by use of a fixed-effects (FE) model (Mantel-Haenszel method) if I^2^≤50%. If I^2^>50%, we tried to find clinical heterogeneity across studies first and addressed it by sensitivity analyses or subgroup analyses, and if significant heterogeneity remained, a random-effects (RE) model would be used if appropriate. To avoid over-estimating a possible effect due to the exclusion of patients in studies with no events in both arms, we performed a reanalysis by adding the reciprocal of one arm size, called as “treatment arm continuity corrections”, to each cell of the opposite arm in trials with zero event [12] using standard software packages STATA version 12.0. For the outcome of TIMI flow grade ≤2, we planned to perform subgroup analyses of studies assessing intracoronary administration of nicorandil and intravenous alone before this systematic review was performed.

## Results

### Study selection

**Figure 1:**
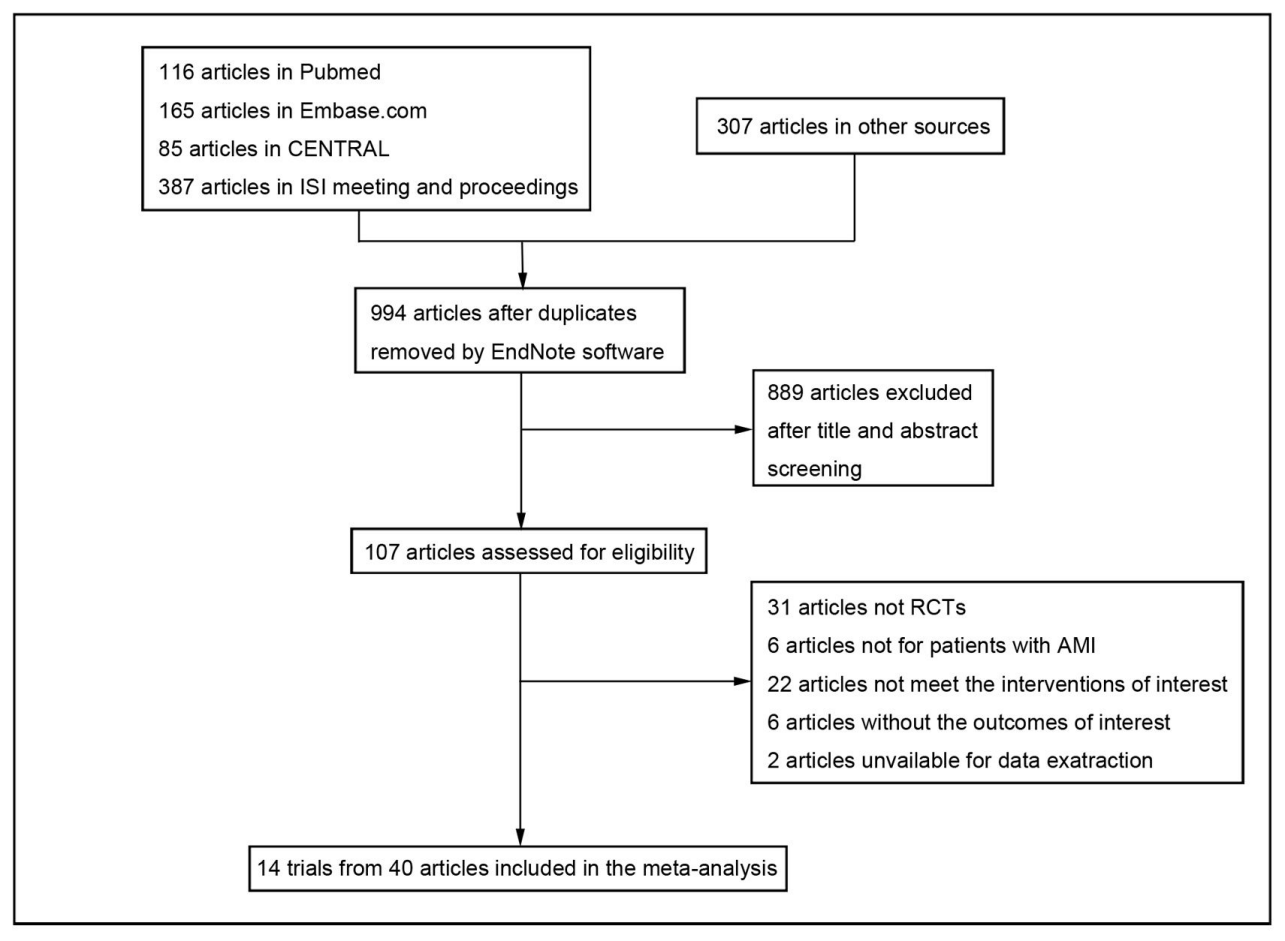
Flow Diagram for Inclusion of Studies in Meta-analysis. The initial search identified 994 articles, of which, 14 trials were included in the final analysis. AMI = acute myocardial infarction; RCTs = randomized controlled trials.

We identified 994 articles after duplicates removed for screening. Based on title and abstract, 889 were excluded and 107 full articles retrieved. 31 were not RCTs and another 36 were excluded for other reasons leaving 40 articles reporting 14 trials that met the inclusion criteria [8-10,13-24]. The process with reasons for exclusion was described in Figure **1**.

### Study characteristics and study quality

**Figure 2:**
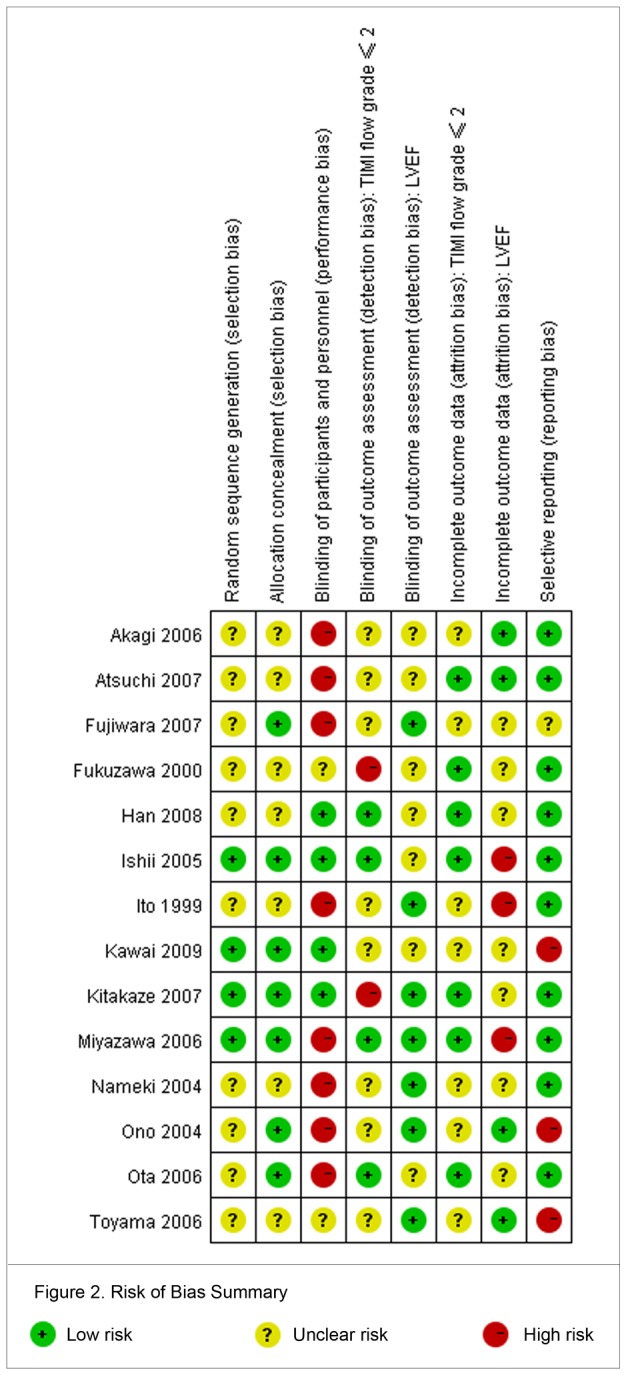
Risk of Bias Summary.

**Figure 3:**
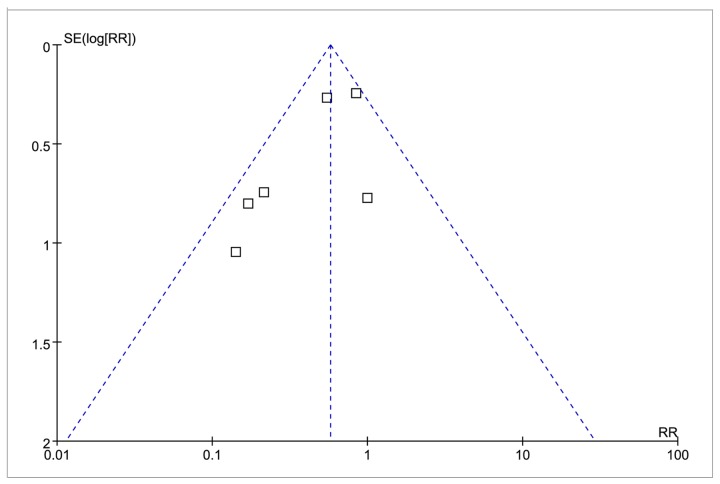
Funnel Plot for the Incidence of TIMI Flow Grade ≤ 2. RR = risk ratio; SE = standard error.

The 14 RCTs involved 1680 patients (range 27 to 545 patients per trial). Table **1** summarized the baseline characteristics, and Table **2** summarized the intervention characteristics of the included trials. One trial [22] was published in abstract, one [15] was a subgroup analysis, and remaining 12 trials have full-text publication. All trials included patients with ST-segment elevation myocardial infarction (STEMI) but one [20] did not report. Patients received nicorandil by intracoronary injection in seven trials, followed by oral nicorandil in four trials, and had reperfusion at mean duration from onset ranging from 3.7 to 7.0 hours in 13 trials. Overall, the quality of the randomized trials included was judged partially limited by a lack of transparency in the reporting of the generation of random sequence generation, for which 4 trials were at low risk, whereas 10 at unclear risk, and in the reporting of the allocation concealment, for which 7 trials were at low risk, whereas 7 at unclear risk. Bias risk assessment of other domains sees details in Figure **2**. In addition, we evaluated the publication bias risk using a funnel plot in Figure **3** based on the outcome TIMI flow grade ≤2.

**Table 1:** Baseline characteristics of included trials.

**Trials**	**Publication type**	**Sample size (NG/CG)**	**Age, mean (SD)**	**Sex (M/F**)	**STEMI or non-STEMI**	**DM, %**	**Occlusion of LAD, %**
**Akagi 2006** [20]	full-text	20/10^#^	64.0 (11.4)	20/10	NR	NR	100
**Atsuchi 2007** [22]	abstract	41/41	NR	NR	STEMI	NR	NR
**Fujiwara 2007** [23]	full-text	31/31	62.0 (10.9)	50/12	STEMI	38.7	50.0
**Fukuzawa 2000** [18]	full-text	31/31	61.4 (12.2)	45/17	STEMI	30.6	64.5
**Han 2008** [16]	full-text	37/36	58.3 (12.6)	61/12	STEMI	31.5	54.8
**Ishii 2005** [8,24]	full-text	185/183	63.5 (9.8)	298/70	STEMI	32.3	47.3
**Ito 1999** [17]	full-text	40/41	60 (9.9)	64/17	STEMI	27.2	100.0
**Kawai 2009** [15]	subgoup-analysis	27/37	NR	NR	STEMI	NR	NR
**Kitakaze 2007** [10]	full-text	276/269	62.4 (10.9)	466/79	STEMI	34.1	49.3
**Miyazawa 2006** [13]	full-text	35/35	62.0 (9.2)	57/13	STEMI	34.3	60.0
**Nameki 2004** [19]	full-text	13/14	63.0 (10.2)	22/7	STEMI	25.9	100.0
**Ono 2004** [9]	full-text	33/25	64.9 (12.4)	38/20	STEMI	32.6	63.8
**Ota 2006** [21]	full-text	63/27^#^	62.8 (10.5)	72/18	STEMI	30.0	46.7
**Toyama 2006** [14]	full-text	33/35	64.0 (12.0)	44/24	STEMI	29.4	60.3

CG = control group; DM = diabetes mellitus; LAD = Left anterior descending coronary artery; M/F = male/female; NG = nicorandil group; NR = not reported; STEMI = ST segment elevation myocardial infarction. ^#^ combining two groups into NG.

**Table 2:** Intervention characteristics of included trials.

**Trials**	**Intervention**	**Subsequent oral nicorandil**	**Combined nitrate**	**Time to reperfusion, mean (SD), hr**	**Rate of tenting, %**
**Akagi 2006** [20]	NG: NIC IV drip infusion (4 mg/hr) for 48 hr, 2 mg IC, and/not 15 mg/day PO. CG: no NIC administered.	Yes	Yes	3.7 (1.7)	NR
**Atsuchi 2007** [22]	NG: NIC 4 mg IV and/or 2 mg IC, IV drip infusion (6 mg/hr) for 24 hr. CG: no NIC administered.	No	NR	NR	NR
**Fujiwara 2007** [23]	NG: NIC 4 mg IV, IV drip infusion (8 mg/hr) for 24 hr. CG: no NIC administered.	No	NR	5.9 (0.6)	NR
**Fukuzawa 2000** [18]	NG: NIC 4 mg IV, IV drip infusion (6 mg/hr) for 24 hr. CG: placebo.	No	Yes	4.6 (2.2)	0
**Han 2008** [16]	NG: NIC 2 mg IC, 2 mg IC. CG: no NIC administered.	No	Yes	5.9 (2.5)	87.7
**Ishii 2005** [8,24]	NG: NIC 12 mg IV over 20-30 min. CG: Placebo.	No	Yes	4.7 (2.9)	82.6
**Ito 1999** [17]	NG: NIC 4 mg IV, IV drip infusion (6 mg/hr) for 24 hr, 15 mg/day PO. CG: no NIC administered.	Yes	Yes	5.1 (2.2)	NR
**Kawai 2009** [15]	NG: NIC 6 mg IV. CG: Placebo.	No	NR	NR	100.0
**Kitakaze 2007** [10]	NG: NIC 0.067 mg/kg IV, IV drip infusion (1.67 μg/kg per min) for 24 hr. CG: placebo.	Yes ^§ ‡^	Yes	3.5 (0.6)	67.9
**Miyazawa 2006** [13]	NG: NIC 2 mg IC, IV drip infusion (2 mg/hr) for 24 hr, 15 mg/day PO. NG: no NIC administered.	Yes	Yes	7.0 (5.2)	82.9
**Nameki 2004** [19]	NG: NIC 4 mg IV, 4 mg IC, IV drip infusion (4 mg/hr) for 24 hr. CG: no NIC administered.	No	Yes	6.3 (4.7)	25.9
**Ono 2004** [9]	NG: NIC 4 mg IV, IV drip infusion (8 mg/hr) for 24 hr. CG: no NIC administered.	No	Yes	5.4 (2.2)	100.0
**Ota 2006** [21]	NG: NIC 1-2 mg IC, and/not IV drip infusion (6 mg/hr) for 16 hr. CG: no NIC administered.	No	Yes	4.0 (1.5)	78.9
**Toyama 2006** [14]	NG: NIC 4 mg IV, 2 mg IC, IV drip infusion (4 mg/hr) for 24 hr. CG: no NIC administered.	No	Yes	5.0 (3.6)	NR

IC = intracoronary; IV = intravenous; NIC = nicorandil; PO = per os.

^§^ both arms received subsequent oral nicorandil; ^‡^ significant difference in patients receiving nicorandil orally between two arms (P <0.05).

### TIMI flow grade

**Figure 4:**
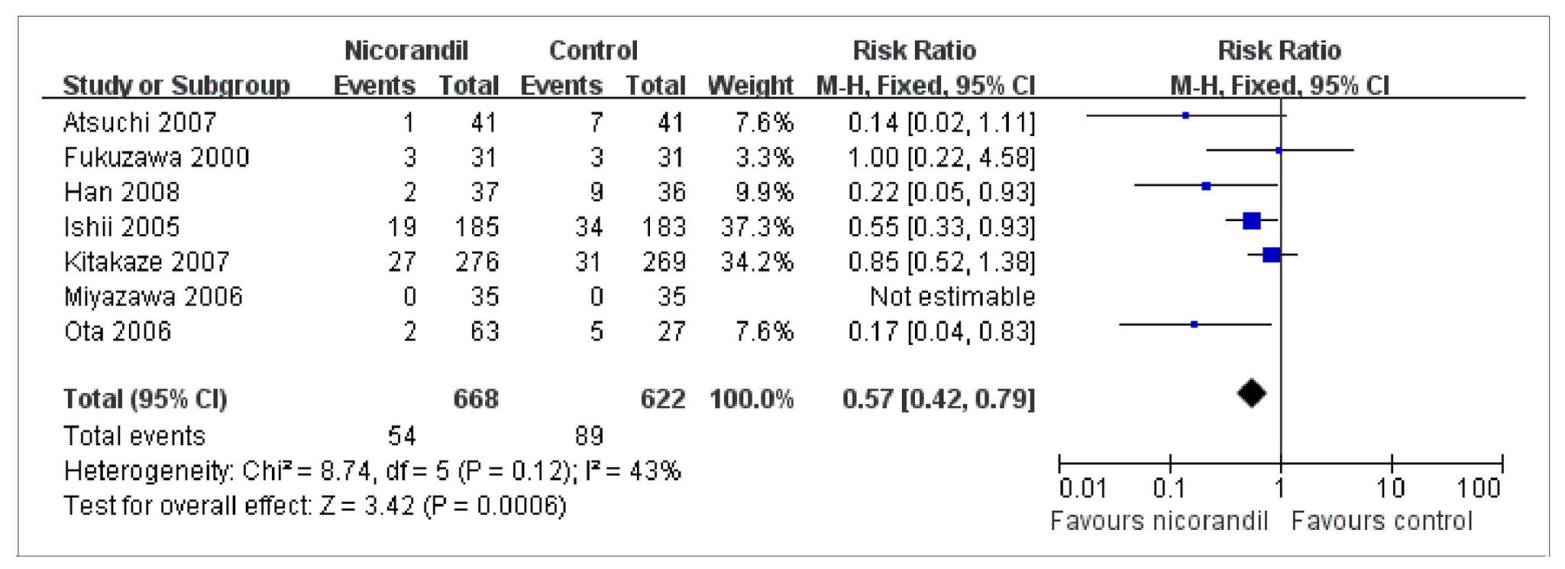
RR of the Incidence of TIMI Flow Grade ≤ 2. Forest plot of RR (with 95% CI) for TIMI flow grade ≤ 2 in patients receiving nicorandil compared with those receiving no nicorandil. Significant reduction in TIMI flow grade ≤ 2 (RR: 0.57; 95% CI: 0.42 to 0.79; p=0.0006) was observed in nicorandil group. CI = confidence interval; RR = risk ratio; TIMI = thrombolysis in myocardial infarction.

After coronary reperfusion, the events of the TIMI flow grade ≤2 occurred in 54 of 668 patients (8.1%) treated with nicorandil and in 89 of 622 patients (14.3%) without nicorandil treatment from seven trials (Figure **4**). Use of nicorandil was associated with a significant reduction in the events of the TIMI flow grade ≤2 (RR, 0.57; 95% CI: 0.42 to 0.79; I^2^=43%; FE model; NNT, 16). The reanalysis including the trial [13] with no event on both arms did not change the result (p=0.002). 

### TFC

**Figure 5:**
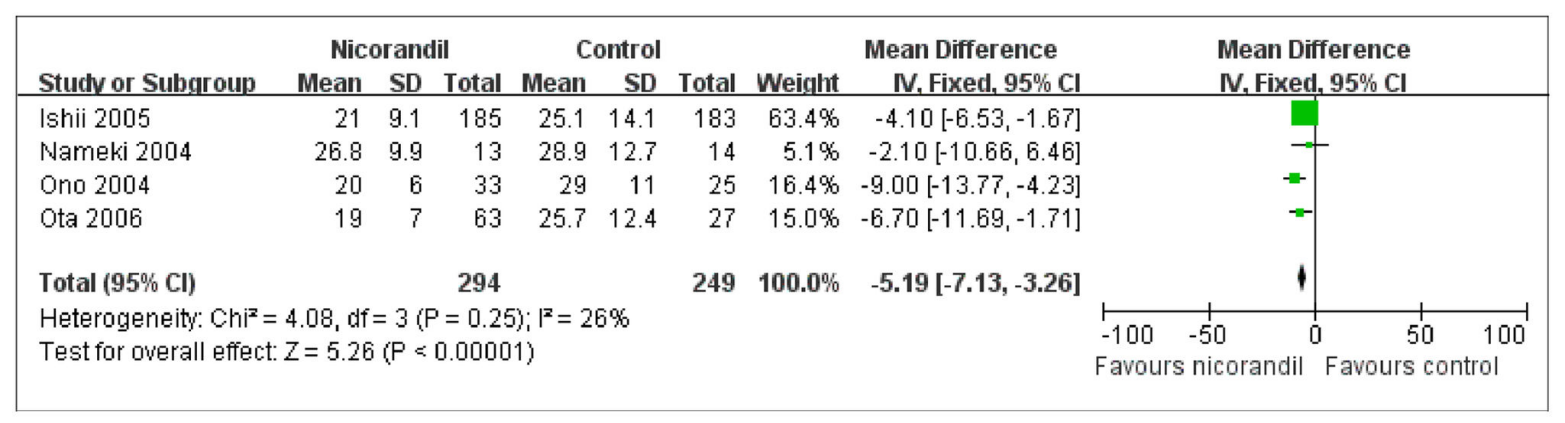
MD of TIMI Frame Count. Forest plot of MD (with 95% CI) for TIMI frame count in patients receiving nicorandil compared with those receiving no nicorandil. Significant reduction in TIMI frame count (MD: -5.19; 95% CI: -7.13 to -3.26; p<0.00001) was observed in nicorandil group. CI = confidence interval; MD = mean difference, TIMI = thrombolysis in myocardial infarction.

The TFC was assessed in 543 patients after reperfusion therapy (Figure **5**). Its value was significantly lower in nicorandil group (MD, -5.19; 95% CI: -7.13 to -3.26; I^2^=26%; FE model) than control group.

### LVEF

**Figure 6:**
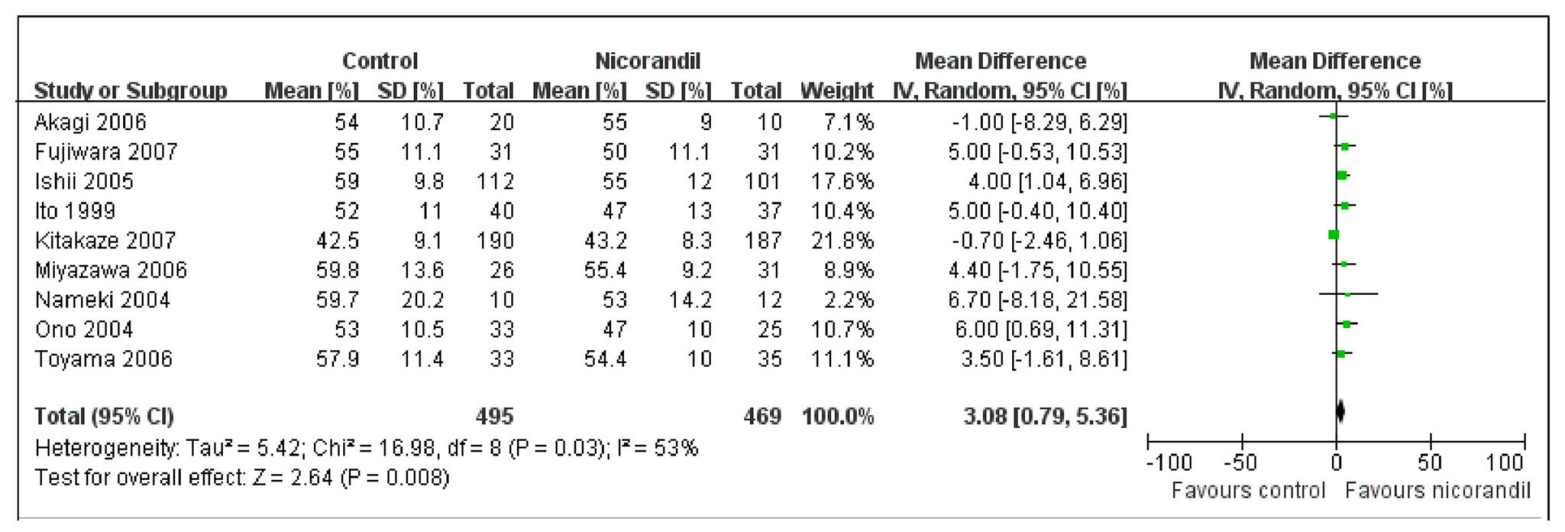
MD of LVEF. Forest plot of MD (with 95% CI) for LVEF in patients receiving nicorandil compared with those receiving no nicorandil. Significant increase in LVEF (MD: 3.08; 95% CI: 0.79 to 5.36; p=0.008) was observed in nicorandil group. CI = confidence interval; LVEF = left ventricular ejection fraction; MD = mean difference.

LVEF was evaluated in 964 patients after one to 12 months (Figure **6**), and using nicorandil was associated with significant increase in LVEF (%) (MD, 3.08; 95% CI: 0.79 to 5.36; I^2^=53%; RF model). A sensitive analysis to exclude one trial [10] found the significant difference remained and heterogeneity reduced (MD, 4.16; 95% CI: 2.34 to 5.97; I^2^=0%; FE model) in the LVEF.

### Peak CK value

The peak CK value was evaluated in 1218 patients from eight trials. But the difference did not reach statistical significance (MD, -290.19; 95% CI: -793.75 to 213.36; I^2^=54%; RE model). 

### VT or VF

Using nicorandil was associated with a significant reduction in the events of ventricular tachycardia or fibrillation within 24 hours or immediate after reperfusion (RR, 0.53; 95% CI: 0.37 to 0.76; I^2^=0%; FE model; NNT, 10.4). The reanalysis including the trial [13] with no event on both arms did not change the result (p=0.001).

### CHF

CHF occurred 11 of 121 patients (9.1%) treated with nicorandil and in 27 of 115 patients treated (23.5%) without nicorandil during hospital. Using nicorandil was associated with a significant reduction in CHF (RR, 0.41; 95% CI: 0.22 to 0.75; I^2^=0%, FE model; NNT, 7.0).

### Cardiac death

Cardiac death was observed 1 of 158 patients (0.6%) in nicorandil group and in 5 of 151 patients (3.3%) in control group. Use of nicorandil was associated with a non-significant reduction in cardiac death (RR, 0.39; 95% CI: 0.09 to 1.67; I^2^=13%; FE model). The reanalysis including the trials with no event on both arms did not change the result (p=0.595).

### Subgroup analysis

As specified, we performed a subgroup analysis separately to assess intracoronary administration of nicorandil and intravenous alone for the outcome TIMI flow grade ≤2, and found nicorandil reduced the events significantly in both subgroups (RR, 0.27; 95% CI: 0.13 to 0.59; I^2^=17%; FE model for the subgroup with intracoronary administration of nicorandil, and RR, 0.69; 95% CI: 0.49 to 0.99; I^2^=28%; FE model for the subgroup with intravenous administration alone).

## Discussion

That is, to the best of our knowledge, the first systematic review and meta-analysis of RCTs that compared nicorandil with no nicorandil or placebo treatment prior to reperfusion in patients with AMI who underwent primary PCI. This systematic review, incorporating more than 1600 patients, showed nicorandil had improved coronary reflow, suppressed reperfusion ventricular arrhythmia, and further improved left ventricular function in this population.

No-reflow phenomenon, which is established at the time of reperfusion as microcirculatory damage, is one of the major causes of impaired functional recovery phase of AMI [25,26]. This analysis showed that administration of nicorandil prior to reperfusion improved the coronary TIMI flow, suppressed ventricular arrhythmia, and further improved left ventricular function. These findings may be due to the effects of nicorandil to suppress reperfusion injury. Nicorandil, as K-ATP channels opener, has potential to dilate resistance arteries with less than 100 µm in diameter [27,28], reduce reactive oxygen species production in cardiac mitochondria at reoxygenation [29], and attenuate ischemia/reperfusion induced polymorphonuclear leukocytes activation via donation of nitric oxide and potassium channel-related cascade [30].

Angiographic microvascular obstruction was defined as TIMI flow grade ≤2 or 3 with a myocardial blush grade <2 in recent studies [31]. In contrast, TIMI flow grade ≤2 and TIMI frame count were used to reflect no/slow reflow in this meta-analysis, which may underestimate the rate of no-reflow events. However, both a retrospective study and a RCT showed that nicorandil reduced the frequency of the no-reflow measured by myocardial contrast echocardiography (MCE) in patients with AMI [17,32], and MCE proves to be a better indicator for assessing microcirculation recovery [33,34].

We have to pay attention to the results of the J-WIND trial [10] which was the largest, multi-center trial among the trials included in this meta-analysis, and found nicorandil treatment could not increase LVEF. However, it was at high risk of incomplete data because of loss of 30% patients at 6 months for measurement of LVEF, and furthermore both arms received oral nicorandil subsequently at the discretion of individual investigators, and the trial showed patients who were given nicorandil orally in the chronic phase had greater increase in LVEF [10] which may be superior to nicorandil treatment prior to reperfusion in a short-term. Further, we performed a sensitive analysis to exclude the trial and found the significant difference remained and heterogeneity was reduced at the endpoint.

Patients with anterior AMI were expected to be at high risk of ventricular arrhythmia, and may have better benefit from ventricular arrhythmia after administration of nicorandil, which may drive the positive result. However, a sensitivity analysis excluding the trial [17] which only enrolled patients with anterior AMI showed significant reduction in ventricular arrhythmia remained (p=0.003). 

There are limitations in the present study. Firstly, one of the major limitations is the small sample size of this meta-analysis, which may give rise to the inconclusive clinical benefit of nicorandil, as we found nicorandil has potential to improve coronary reflow and LVEF and previous studies have shown no or slow coronary reflow and LVEF are the important determinants of prognoses after AMI [35-37]. So additional large scale researches are required to confirm the impact of nicorandil prior to reperfusion on clinical outcomes. Meanwhile, the small sample size might also lower the power to conclude the benefit of these surrogate endpoints. Secondly, a majority of trials in this meta-analysis did not report whether to use random sequence or allocation concealment, thus some trials with high risk bias might be included. Thirdly, this review contained trials regardless of dose of nicorandil, administration routes, duration of follow-up and location of occlusion which may also bring some heterogeneity. Fourthly, we did not perform subgroup analyses to evaluate nicorandil in patients with angina before onset of myocardial infarction, which may mimic ischemic precondition to abolish the effect of nicorandil [38], but few related data was allowed to be pooled. Fifthly, the majority of the trials included are limited to Japan. Finally, we did not show the incidence of side effects of nicorandil since the trials included did not provide related information. Recent evidences showed that nicorandil can induce ulceration [39], but some have postulated that the risk of ulceration is dose dependent [40] , so the dose of nicorandil prior to reperfusion may be not enough to cause ulceration. In addition, a small study found that high-dose nicorandil therapy (bolus intravenous injection 0.2mg/kg followed by 0.2mg/kg/h for ≥24h) prior to reperfusion could be completed safely without any increase in hypotension, fatal arrhythmia, or nicorandil discontinuation in patients with AMI compared to standard dose nicorandil therapy (infusion at 0.2mg/kg/h for ≥24h) [41].

## Conclusions

Nicorandil prior to reperfusion is associated with improvement of coronary reflow as well as suppression of reperfusion ventricular arrhythmia, and further improves left ventricular function in patients who suffered from AMI and underwent primary PCI. But the definite clinical benefits of nicorandil were not found, which may be due to the small sample size of the selected studies. Additional large scale researches are required to confirm the impact of nicorandil on clinical outcomes.

## Supporting Information

Table S1 **Search Strategy.**
(DOC)

Checklist S1 **PRSIMA 2009 Statement Checklist.**
(DOC)
