# Supplementary material for: Nicorandil in Patients with Acute Myocardial Infarction Undergoing Primary Percutaneous Coronary Intervention: A Systematic Review and Meta-Analysis
Source: PLoS One. 2013 Oct 22;8(10):e78231. doi: 10.1371/journal.pone.0078231 (PMC3805586; doi:10.1371/journal.pone.0078231)
Supplement: Table S1 — Search Strategy. (DOC) [file pone.0078231.s001.doc]

| **Table S1.** Search Strategy | | |
| --- | --- | --- |
| **CENTRAL** | | |
| **#1** | MeSH descriptor: [Nicorandil] explode all trees | 122 |
| **#2** | nicorandil in Trials (Word variations have been searched) | 170 |
| **#3** | Sigmart in Trials (Word variations have been searched) | 7 |
| **#4** | Ikorel in Trials (Word variations have been searched) | 1 |
| **#5** | Dancor in Trials (Word variations have been searched) | 0 |
| **#6** | Nikoran in Trials (Word variations have been searched) | 0 |
| **#7** | Angedil in Trials (Word variations have been searched) | 0 |
| **#8** | Nitorubin in Trials (Word variations have been searched) | 0 |
| **#9** | 2 nicotinamidethyl nitrate in Trials (Word variations have been searched) | 1 |
| **#10** | 2 Nicotinamidoethyl Nitrate in Trials (Word variations have been searched) | 0 |
| **#11** | SG75 or 'SG 75' or SG-75 in Trials (Word variations have been searched) | 305 |
| **#12** | (k or k? or potassium) near channel near opener in Trials (Word variations have been searched) | 107 |
| **#13** | #1 or #2 or #3 or #4 or #5 or #6 or #7 or #8 or #9 or #10 or #11 or #12 | 534 |
| **#14** | MeSH descriptor: [Myocardial Infarction] explode all trees | 7936 |
| **#15** | myocard* near infarct* in Trials (Word variations have been searched) | 12465 |
| **#16** | heart near infarct* in Trials (Word variations have been searched) | 2878 |
| **#17** | cardiac near infarct* in Trials (Word variations have been searched) | 1047 |
| **#18** | ST next segment next elevation in Trials (Word variations have been searched) | 993 |
| **#19** | non next ST next segment next elevation in Trials (Word variations have been searched) | 227 |
| **#20** | STEMI in Trials (Word variations have been searched) | 421 |
| **#21** | non next STEMI in Trials (Word variations have been searched) | 7 |
| **#22** | myocard* near necrosis in Trials (Word variations have been searched) | 159 |
| **#23** | #14 or #15 or #16 or #17 or #18 or #19 or #20 or #21 or #22 in Trials (Word variations have been searched) | 12956 |
| **#24** | #13 and #23 in Trials (Word variations have been searched) | 85 |
| **PUBMED** | | |
| **#1** | "Nicorandil"[Mesh] | 1105 |
| **#2** | Nicorandil[tw] | 1328 |
| **#3** | Ikorel[tw] | 5 |
| **#4** | Dancor[tw] | 1 |
| **#5** | Nikoran[tw] | 1 |
| **#6** | Sigmart[tw] | 12 |
| **#7** | "2 Nicotinamidoethyl Nitrate"[tw] | 34 |
| **#8** | "2 nicotinamidethyl nitrate"[tw] | 8 |
| **#9** | SG75[tw] OR “SG 75”[tw] OR “SG-75” | 65 |
| **#10** | #1 OR #2 OR #3 OR #4 OR #5 OR #6 OR #7 OR #8 OR #9 | 1350 |
| **#11** | (k[tw] OR k+[tw] OR potassium[tw]) AND channel[tw] AND opener[tw] | 2206 |
| **#12** | #10 OR #11 | 3296 |
| **#13** | "Myocardial Infarction"[Mesh] exp | 138126 |
| **#14** | myocard*[tw] AND infarct*[tw] | 185179 |
| **#15** | heart[tw] AND infarct*[tw] | 77852 |
| **#16** | cardiac[tw] AND infarct*[tw] | 58755 |
| **#17** | st segment elevation[tw] | 8197 |
| **#18** | non st segment elevation[tw] | 1554 |
| **#19** | STEMI[tw] | 3669 |
| **#20** | non STEMI[tw] | 194 |
| **#21** | myocardi*[tw] AND necrosis[tw] | 13066 |
| **#22** | #13 OR#14 OR #15 OR #16 OR #17 OR #18 OR #19 OR #20 OR #21 | 204518 |
| **#23** | #12 AND #22 | 375 |
| **#24** | randomized controlled trial[pt] | 336388 |
| **#25** | controlled clinical trial[pt] | 84881 |
| **#26** | randomized[tiab] | 277878 |
| **#27** | placebo[tiab] | 145437 |
| **#28** | drug therapy[sh] | 1562712 |
| **#29** | randomly[tiab] | 190283 |
| **#30** | trial[tiab] | 319645 |
| **#31** | groups[tiab] | 1249453 |
| **#32** | #26 OR #27 OR #28 OR #29 OR #30 OR #31 OR #32 OR #33 | 3088850 |
| **#33** | animals[mh] | 16219056 |
| **# 34** | humans[mh] | 12478860 |
| **#35** | #33 NOT #34 | 3740196 |
| **#36** | #32 NOT #35 | 2647412 |
| **#37** | #36 AND #23 | 116 |
| **EMBASE.com** | | |
| **#1** | Nicorandil.mp. or nicorandil/ | 3093 |
| **#2** | Nicorandil.tw. | 1680 |
| **#3** | Ikorel.tw. | 60 |
| **#4** | Dancor.tw. | 17 |
| **#5** | Nikoran.tw. | 4 |
| **#6** | Sigmart.tw. | 32 |
| **#7** | Angedil.tw. | 0 |
| **#8** | Aprior.tw. | 2 |
| **#9** | Nitorubin.tw. | 0 |
| **#10** | 2 Nicotinamidoethyl Nitrate.tw. | 44 |
| **#11** | 2 nicotinamidethyl nitrate.tw. | 11 |
| **#12** | SG75.tw. | 3 |
| **#13** | "SG 75".tw. | 123 |
| **#14** | SG-75.tw. | 123 |
| **#15** | ((k or k+ or potassium) adj3 channel adj3 opener).tw. | 1757 |
| **#16** | 1 or 2 or 3 or 4 or 5 or 6 or 7 or 8 or 9 or 10 or 11 or 12 or 13 or 14 or 15 | 4552 |
| **#17** | myocardial infarction.mp. or heart infarction/ | 265479 |
| **#18** | (myocard$ adj7 infarct$).tw. | 182369 |
| **#19** | (heart adj7 infarct$).tw. | 18309 |
| **#20** | (cardiac adj7 infarct$).tw. | 17083 |
| **#21** | (myocard$ adj7 necrosis).tw. | 5918 |
| **#22** | "ST segment elevation".tw. | 1273 |
| **#23** | "non ST segment elevation".tw. | 2112 |
| **#24** | STEMI.tw. | 7821 |
| **#25** | "non STEMI".tw. | 389 |
| **#26** | 17 or 18 or 19 or 20 or 21 or 22 or 23 or 24 or 25 | 277757 |
| **#27** | crossover procedure/ | 35655 |
| **#28** | double blind procedure/ | 114544 |
| **#29** | controlled clinical trial/ | 393369 |
| **#30** | single blind procedure/ | 16704 |
| **#31** | random$.tw. | 781902 |
| **#32** | factorial$.tw. | 20376 |
| **#33** | crossover$.tw. | 45895 |
| **# 34** | cross over$.tw. | 20905 |
| **#35** | placebo$.tw. | 187774 |
| **#36** | (double$ adj blind$).tw. | 139040 |
| **#37** | (single$ adj blind$).tw. | 13104 |
| **#38** | assign$.tw. | 217266 |
| **#39** | allocat$.tw. | 73578 |
| **#40** | volunteer$.tw. | 168464 |
| **#41** | 27 or 28 or 29 or 30 or 31 or 32 or 33 or 34 or 35 or 36 or 37 or 38 or 39 or 40 | 1379169 |
| **#42** | 16 and 26 and 41 | 165 |
| **ISI meeting and proceeding** | | |
| **1#** | Topic=(Nicorandil) OR Topic=(Ikorel) OR Topic=(Dancor) OR Topic=(Nikoran) OR Topic=(Sigmart) OR Topic=(Angedil) OR Topic=(Aprior) OR Topic=(Nitorubin) OR Topic=(“2 Nicotinamidoethyl Nitrate”) OR Topic=(“2 nicotinamidethyl nitrate”) OR Topic=(SG75) OR Topic=(SG-75) OR Topic=(“SG 75”) OR Topic=((k OR k+ OR potassium) SAME channel SAME opener)  Databases=CPCI-S Timespan=All Years  Lemmatization=On | 387 |
| **CBM** | | 55 |
| **Google Scholar** | | 160 |
| **Supplements of seven international core journals** | |  |
| Supplements of Circulation | | 23 |
| Supplements of JACC | | 21 |
| Supplements of European Heart Journal | | 18 |
| Supplements of Heart | | 3 |
| Supplements of American Heart Journal | | 5 |
| Supplements of American Journal of Cardiology | | 4 |
| Supplements of International Journal of Cardiology | | 3 |
| **TCTMD** | | 6 |
| **Clinical trial register** | | 9 |
| **Reference lists of relevant articles** | | 0 |
| **Total reports** | | 1060 |
| **After de-duplicated by Endnote** | | 994 |
